# Enhanced Solubility and Electron Transfer of Osmium-Based Mediators via Quaternized Poly(4-Vinylpyridine) for Electrochemical Glucose Detection

**DOI:** 10.3390/polym17212874

**Published:** 2025-10-28

**Authors:** Yun Yeong Cho, Tae-Won Seo, Young-Bong Choi, Won-Yong Jeon

**Affiliations:** 1 Department of Chemistry, College of Science & Technology, Dankook University, 119 Dandae-ro, Cheonan-si 31116, Chungcheongnam-do, Republic of Korea; whuy1220@naver.com (Y.Y.C.); xodnjs7508@dankook.ac.kr (T.-W.S.); 2 Department of Cosmedical Materials, College of Bio-Convergence, Dankook University, 119 Dandae-ro, Cheonan-si 31116, Chungcheongnam-do, Republic of Korea; 3 Graduate School of Management of Technology, Hoseo University, Asan-si 31499, Chungcheongnam-do, Republic of Korea; 4 Fine Dust & Net Zero Research Institute, Hoseo University, Asan-si 31499, Chungcheongnam-do, Republic of Korea; 5 Eco Upcycling R&D Center, Hoseo University, Asan-si 31499, Chungcheongnam-do, Republic of Korea

**Keywords:** electrochemical biosensor, continuous glucose monitoring (CGM), mediator, quaternization

## Abstract

Hydrophilic polymer–osmium complexes enhance electron transfer between enzymes and electrodes in biosensors. In this study, hydrophobic poly(4-vinylpyridine) (PVP) was quaternized with 2-bromoethanol to synthesize water-soluble PVP(Q)-C_2_H_4_OH polymers (MW 60,000 and 160,000). The resulting PVP(Q)-C_2_H_4_OH-Os(dmo-bpy)_2_Cl complexes were verified by UV-Vis, FT-IR, 1H NMR, SEM-EDS, and zeta potential analyses, confirming successful quaternization and osmium coordination with good dispersion stability. Electrochemical tests (cyclic voltammetry, multi-potential step, amperometry) demonstrated that electrodes with quaternized mediators showed greatly enhanced catalytic currents for glucose (0–20 mM), with sensitivities of 6.9791 (MW 60,000) and 6.6279 μA·mM^−1^·cm^−2^ (MW 160,000), respectively, which were 6.6–10.3 times higher than those of non-quaternized polymers. Selectivity tests showed negligible interference from common species such as ascorbic acid, dopamine, uric acid, and serotonin. Continuous glucose monitoring (CGM) electrodes were fabricated by immobilizing the mediator and glucose dehydrogenase on silanized Au electrodes. SEM, scan rate, and impedance analyses confirmed stable binding. The modified electrodes showed strong linearity (R^2^ = 0.992) and high sensitivity (2.56 μA·mM^−1^·cm^−2^), and good stability, maintaining ~82% activity for seven days. Human plasma testing validated accurate glucose detection (6.05 mM), consistent with physiological levels. Overall, quaternized PVP(Q) mediators significantly improved solubility and electron transfer, enabling the development of a stable, selective glucose sensor suitable for CGM applications.

## 1. Introduction

Diabetes is a chronic metabolic disorder caused by insufficient insulin secretion or ineffective insulin utilization, leading to elevated blood glucose that can damage major organs. Although incurable, maintaining stable glucose levels reduces complications, making accurate and continuous glucose monitoring (CGM) technologies increasingly vital [1,2,3].

A biosensor detects biological changes and converts them into measurable signals. Among these, blood glucose sensors are the most representative, assessing glucose levels for diagnosis and management of diabetes. Key performance indicators include sensitivity, response time, selectivity, stability, and user convenience [4,5,6]. For efficient diagnosis and continuous monitoring, glucose sensors must provide accurate and repeated measurements. Depending on the signal transducer, biosensors are classified into optical, piezoelectric, thermal, and electrochemical type [7].

Piezoelectric, optical, and thermal biosensors each have advantages but suffer from limitations such as reduced sensitivity in liquid system, difficulty in detecting minor changes, and poor performance in low-heat reactions [8,9,10]. Electrochemical biosensors, however, offer high sensitivity, fast response, miniaturization, and low cost, making them the most widely used [11]. Enzyme-based electrochemical glucose sensors, especially those using glucose oxidase (GOx), show high selectivity and stability under varying pH and temperature [12].

Electrochemical glucose sensors have evolved through three generations. The first generation relies on oxygen as a mediator, but its current output is limited by oxygen concentration, and substitution with glucose dehydrogenase (GDH) reduces this dependence but maintains low electron transfer efficiency [13,14,15]. The second generation introduces mediators such as ferrocene, ferrocyanide, ruthenium, and osmium complexes to facilitate faster electron transfer [13].

Among these mediators, ferrocene derivatives have been widely explored due to their reversible redox behavior and structural tunability; however, their hydrophobicity and instability in aqueous media often restrict long-term biosensor performance. Recent studies, such as the hybridization chain reaction (HCR)-based electrochemical biosensor reported by Xia et al. [16], have demonstrated that integrating redox mediators into hybridized homogeneous–heterogeneous systems can enhance signal amplification and mediator accessibility, ultimately improving electron-transfer kinetics. These findings emphasize the importance of designing hydrophilic and structurally stable redox mediators for continuous glucose monitoring (CGM) applications.

These have led to two practical systems: blood glucose monitoring (BGM), which measures blood glucose directly, and CGM, which detects glucose in interstitial fluid. BGM offers simplicity and affordability but requires repeated blood sampling, whereas CGM enables real-time, long-term monitoring without invasive sampling, despite higher cost [17,18].

The third-generation directly fixes the enzyme to the electrode, removing the need for mediators, but mass production remains difficult [19]. CGM biosensors must detect low glucose concentrations in biological fluids such as tears, saliva, urine, and sweat, necessitating highly sensitive and stable conductive materials [20].

Conductive polymers such as poly(4-vinylpyridine) (PVP) are attractive for electrochemical applications due to their doping ability and conductivity; however, their limited solubility hinders use as mediators. Thus, studies have focused on synthesizing water-soluble polymers through copolymerization or quaternization of PVP [21,22].

In this study, hydrophobic PVP was quaternized to form a hydrophilic polymer, and a novel osmium complex was synthesized as an electron transfer mediator for CGM. The synthesized compound was characterized by UV-vis, FT-IR, SEM-EDS, and ^1^H-NMR analyses, while electrochemical properties were evaluated via cyclic voltammetry (CV), multi-potential step (MPS), zeta potential, and amperometric I-t curves. The developed mediator exhibited excellent dispersibility and long-term stability, demonstrating its strong potential for next-generation CGM sensor applications.

## 2. Materials and Methods

### 2.1. Chemicals and Reagents

4,4′-Dimethoxy-2,2′-bipyridine, ammonium persulfate, 2-bromoethanol, poly(4-vinylpyridine) (MW 160,000/60,000), poly(ethylene glycol) diglycidyl ether (PEGDGE), glutaraldehyde solution, isopropyl alcohol (IPA), D-(+)-glucose, ascorbic acid (AA), dopamine hydrochloride (DA), uric acid (UA), serotonin, 2-mercaptoethanol, and (3-glycidyloxypropyl)trimethoxysilane (3-GOPTS) were purchased from Sigma–Aldrich Co., Ltd. (Milwaukee, WI, USA). Ammonium hexachloroosmate (IV) was obtained from Alfa Aesar Co., Ltd. (Ward Hill, MA, USA). The enzyme glucose dehydrogenase (FAD-dependent GDH, 589 U/mg) was supplied by Toyobo Co., Ltd. (Osaka, Japan). N,N-dimethylformamide, ethyl ether, methanol, and ethanol were acquired from Daejung Chemicals & Metals Co., Ltd. (Siheung, Republic of Korea) and used as received. Toluene was obtained from Samchun Chemicals & Metals Co., Ltd. (Pyeongtaek, Republic of Korea). Human Plasma-Like Medium was procured from Thermo Fisher Scientific Co., Ltd. (Waltham, MA, USA). All materials were used as received (GR grade).

### 2.2. Instrument

Electrochemical measurements were carried out using a CHI 660B electrochemical workstation (CH Instruments, Inc., Austin, TX, USA). FT-IR spectroscopy (Cary 630, Agilent Technologies, Shanghai, China) and ^1^H-NMR spectroscopy (Bruker Avance III 400, 400 MHz, Billerica, MA, USA) were used to confirm the synthesis of the electron transfer mediator. The particle dispersion of the synthesized electron transfer mediator was analyzed using a zeta potential analyzer (SZ-100, Horiba, Fuchu, Hiroshima, Japan). Field-emission SEM (FE-SEM, SIGMA 500, ZEISS, Oberkochen, Germany) was used to characterize the immobilization on the electrode surface.

### 2.3. Synthesis of Mediators and Polymers

Synthesis of Os(dmo-bpy)_2_Cl_2_: Os(dmo-bpy)_2_Cl_2_ was synthesized using a modified procedure from the literature [22]. Ammonium hexachloroosmate (700 mg, 1.59 mmol) and 4,4′-dimethoxy-2,2′-bipyridine (758.53 mg, 3.51 mmol) were heated in 70 mL of ethylene glycol at 160 °C under reflux for 50 min. Sodium hydrosulfite (11.1 g, 63.8 mmol) in 200 mL of distilled water was added, and the mixture was maintained at 4 °C for 2 h. The precipitate was filtered through a 0.45 μm nylon membrane and dried at 40 °C for 48 h.

Synthesis of PVP(Q)-C_2_H_4_OH: PVP (2 g, 19.02 mmol) and 2-bromoethanol (0.4754 g, 3.80 mmol) were dissolved in 60 mL of DMF and heated to 85–90 °C under reflux in a nitrogen atmosphere for 24 h. The product was precipitated in 600 mL of ethyl ether and dried in a vacuum oven at 40 °C for 24 h [23].

Synthesis of PVP(Q)-C_2_H_4_OH-Os(dmo-bpy)_2_Cl (MW 60,000 and 160,000): Os(dmo-bpy)_2_Cl_2_ (181.2 mg for MW 60,000; 171.5 mg for MW 160,000) and PVP(Q)-C_2_H_4_OH polymers (137.4 mg for MW 60,000; 130 mg for MW 160,000) were dissolved in 60 mL of ethanol and heated to 120 °C under reflux for 9 h. The crude product was precipitated in 600 mL of ethyl ether and dried (40 °C, 24 h). The residue was dissolved in 50 mL of distilled water and purified using a 10 kDa ultrafiltration membrane. The solution was reprecipitated in ethyl ether and oven-dried.

Synthesis of PVP-Os(dmo-bpy)_2_Cl (MW 60,000 and 160,000): Os(dmo-bpy)_2_Cl_2_ (102.3 mg for MW 60,000; 155.6 mg for MW 160,000) and PVP (77.5 mg for MW 60,000; 117.9 mg for MW 160,000) were dissolved in 50 mL of ethanol and heated to 120 °C under reflux for 9 h. The solid was precipitated in ethyl ether, dried, and partially redissolved in an ethanol/water mixture owing to its limited solubility. Purification was conducted using a 10 kDa ultrafiltration membrane, followed by reprecipitation and drying [24].

### 2.4. Fabrication of Electrodes

SPCEs: Screen-printed carbon electrodes (SPCEs, 4.0 mm diameter) were fabricated by printing carbon ink (LOCTITE^®^ EDAG 965SS E&C, Henkel, Germany) onto OHP films using a semi-automatic screen printer (BS-860-AP, Bando, Pusan, Republic of Korea).

Au electrode pretreatment: Au electrodes (Nemesis, Seoungnam, Republic of Korea) were cleaned by immersion in toluene for 24 h, rinsed with methanol followed by distilled water, and dried under argon.

Silane modification of Au electrodes: Au electrodes were immersed in a 10 mM 2-mercaptoethanol solution for 24 h to activate the hydroxyl groups. After rinsing with ethanol and water, the electrodes were dried with argon and immersed in 4% (*v*/*v*) 3-GOPTS solution for 24 h, followed by IPA washing and argon drying.

**Scheme 1:**
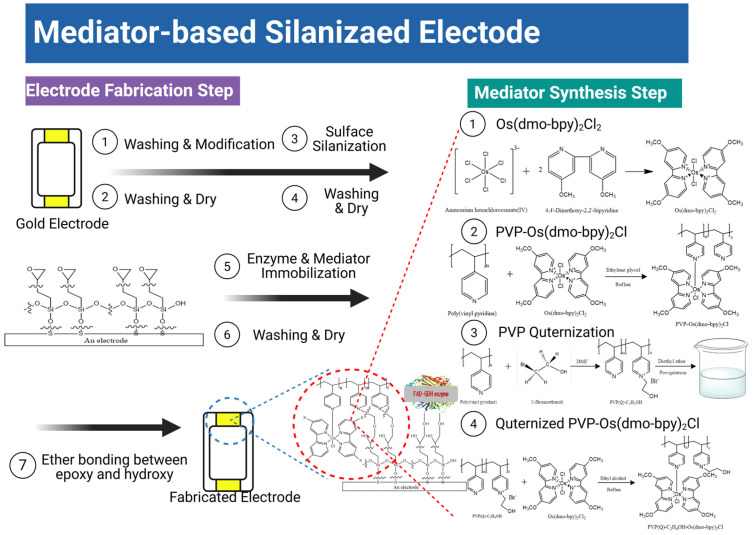
Mediator synthesis and electrode fabrication steps leading to quaternized PVP-Os-based electrodes.

Mediator/GDH immobilization: For SPCEs, mediator/GDH/PEGDGE mixtures (4:4:1 *v*/*v*) were prepared using mediator (10 mg/mL), GDH (40 mg/mL), and PEGDGE (10 mg/mL). A 10 μL aliquot was drop-cast on SPCEs, dried at 30 °C for 30 min, and stored in a desiccator for 24 h. For the silane/Au electrodes, mediator/GDH/PEGDGE mixtures (1 mg/mL mediator, 10 mg/mL GDH, 10 mg/mL PEGDGE) were similarly prepared, drop-cast (10 μL), oven-dried (30 °C, 30 min), desiccated (24 h), ultrasonicated in PBS to remove the physisorbed species, and finally dried under nitrogen [25]. The detailed reaction mechanism and corresponding process are summarized in Scheme 1.

## 3. Results

### 3.1. Structural Characterization of the Mediators

Figure S1 shows the FT-IR spectra to confirm the quaternization of PVP. The spectra of the monomer 2-bromoethanol, PVP with different molecular weights, and the quaternized polymer PVP(Q)-C_2_H_4_OH were recorded using the ATR mode and analyzed for the characteristic peaks. In the spectrum of PVP(Q)-C_2_H_4_OH, the shift in the aromatic C=N stretching band to approximately 1640 cm^−1^ suggested that quaternization occurred at the nitrogen atom of the pyridine ring in PVP. In addition, the successful incorporation of 2-bromoethanol into the polymer was confirmed by the appearance of O–H and C–H stretching bands near 3400 cm^−1^ and 3250 cm^−1^, respectively. The disappearance of the C–Br stretching band at approximately 660 cm^−1^, originally present in the 2-bromoethanol monomer, confirmed the successful synthesis of PVP(Q)-C_2_H_4_OH.

Figure S2 presents the ^1^H-NMR spectrum recorded to verify the quaternization of PVP. The spectrum of PVP(Q)-C_2_H_4_OH was obtained in D_2_O and analyzed for characteristic proton signals. The peaks observed in the range of 6–8.5 ppm correspond to the aromatic protons of the pyridine ring, while the signals at 3.5–4.5 ppm were attributed to the alkyl groups derived from 2-bromoethanol. The resonances appearing at 2–3 ppm were assigned to protons on the carbons adjacent to the quaternized nitrogen atom of the pyridine ring. In addition, the signals at 3–4 ppm and 1–5 ppm were assigned to the hydroxymethyl (HO–CH) and hydroxyl (R-OH) protons, respectively. These results confirm that 2-bromoethanol had been introduced into the polymer structure [26].

Although the feed ratio of PVP to 2-bromoethanol was designed as 5:1, the quantitative integration of proton signals in the ^1^H-NMR spectra indicated that the actual incorporation ratio was approximately 4.1:1 for the high- and low-molecular-weight polymers [27].

Figure S3 presents the zeta potential measurements used to evaluate the dispersion stability of PVP(Q)-C_2_H_4_OH-Os(dmo-bpy)_2_Cl. Generally, particles with zeta potentials in the range of −10 to +10 mV are considered electrically neutral, while values below −30 mV and above +30 mV are typically regarded as indicative of anionic and cationic characteristics, respectively. The measured zeta potentials of the synthesized complexes were +90.444 mV and +88.558 mV, showing that the particles exhibit strong cationic properties. These results confirm that PVP(Q)-C_2_H_4_OH-Os(dmo-bpy)_2_Cl possesses excellent dispersion stability, regardless of the molecular weight of the polymer [28].

### 3.2. Electrochemical Properties of Mediators

Figure S4 presents the CV results obtained to verify the successful synthesis of PVP(Q)-C_2_H_4_OH-Os(dmo-bpy)_2_Cl compared to Os(dmo-bpy)_2_Cl_2_. For the measurements, 20 μL of the sample solution and 20 μL of 1× PBS were mixed and applied (total volume: 40 μL) to the SPCEs. In Figure S4, the blue trace corresponds to Os(dmo-bpy)_2_Cl_2_, while the red trace represents PVP(Q)-C_2_H_4_OH-Os(dmo-bpy)_2_Cl.

The redox potential of Os(dmo-bpy)_2_Cl_2_ was observed at −0.24 V, whereas that of PVP(Q)-C_2_H_4_OH-Os(dmo-bpy)_2_Cl shifted to +0.097 V. This positive shift resulted from the electron-withdrawing pyridine groups in the polymer backbone that stabilize the Os complex. These results confirmed the successful synthesis of PVP(Q)-C_2_H_4_OH-Os(dmo-bpy)_2_Cl using the 160,000 MW PVP(Q).

### 3.3. Morphological Properties of Electrodes

Figure S5 presents the SEM and energy dispersive spectroscopy (EDS) analyses performed to validate the surface immobilization and successful synthesis of the PVP(Q)-C_2_H_4_OH-Os(dmo-bpy)_2_Cl-based electrodes. The SEM images revealed the surface morphology of modified SPCEs, while the EDS spectra provided elemental confirmation. The detection of oxygen, originating from the quaternized water-soluble polymer, together with the characteristic osmium signals, showed that osmium had been successfully coordinated to the polymer backbone and immobilized on the electrode surface.

**Figure 1:**
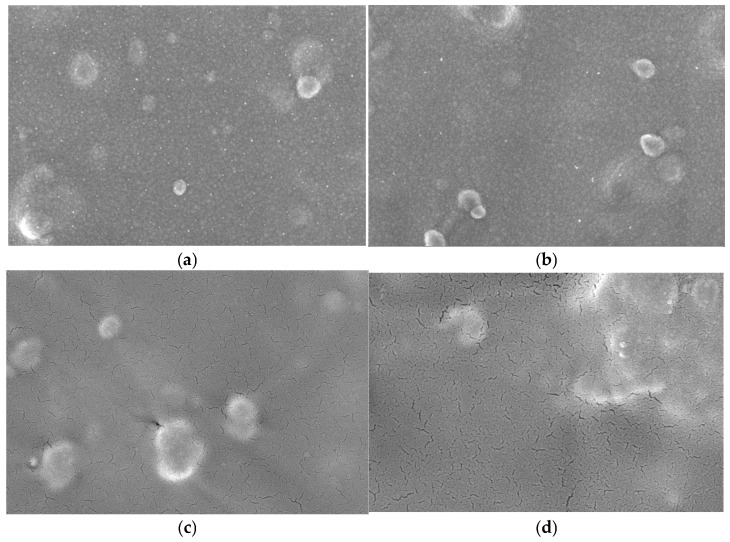
SEM images (×80 K) of (**a**) bare Au electrode, (**b**) Silane/Au electrode, (**c**) Mediator/Silane/Au electrode, (**d**) Mediator/GDH/Silane/Au electrode.

Figure 1 shows the FE-SEM images used to examine the surface morphology of the electrodes. The bare Au electrode displayed multiple impurities on its surface because of the absence of toluene cleaning, as shown in Figure 1a. In contrast, the silanized Au electrode (Figure 1b) exhibited a smoother and more homogeneous surface, confirming successful surface modification.

When GDH was immobilized on the silane-modified Au electrode (Figure 1c), the surface became smoother, indicating the formation of a uniform enzyme layer. Finally, the electrode modified with PVP(Q)-C_2_H_4_OH-Os(dmo-bpy)_2_Cl and GDH (Figure 1d) displayed aggregated clusters, suggesting the formation of large molecular assemblies. These results confirm that the mediator and the enzyme had been successfully immobilized on the Au electrode surface.

### 3.4. Electrochemical Characteristics of PVP(Q)-C_2_H_4_OH-Os(dmo-bpy)_2_Cl/GDH/SPCEs

**Figure 2:**
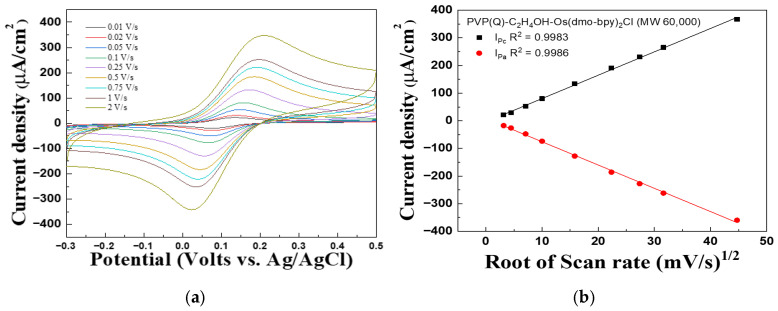
(**a**) Cyclic voltammograms and (**b**) Calibration curve vs. the square root of the scan rate for the PVP(Q)-C_2_H_4_OH-Os(dmo-bpy)_2_Cl (MW 60,000).

**Figure 3:**
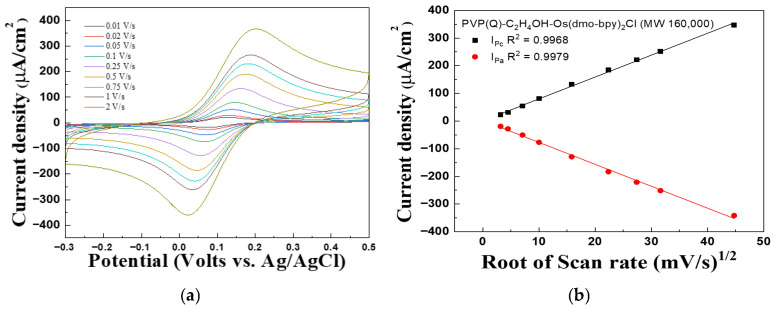
(**a**) Cyclic voltammograms and (**b**) Calibration curve vs. square root of the scan rate for the PVP(Q)-C_2_H_4_OH-Os(dmo-bpy)_2_Cl (MW 160,000).

Figure 2 and Figure 3 present the CV graph of PVP(Q)-C_2_H_4_OH-Os(dmo-bpy)_2_Cl/GDH/SPCEs with different molecular weights, recorded at scan rates ranging from 0.01 to 2 V/s, to evaluate the performance of the complexes as electron transfer mediators and investigate diffusion effects in solution. In Figure 2b, the linear dependence of the cathodic peak current (I_pc_, R^2^ = 0.9983) and anodic peak current (I_pa_, R^2^ = 0.9986) on the square root of the scan rate was observed. Similarly, I_pc_ (R^2^ = 0.9968) and I_pa_ (R^2^ = 0.9979) exhibited excellent linearity, as shown in Figure 3b.

These results suggest that the current density is proportional to the square root of the scan rate, suggesting that the electrochemical behavior of the electrodes is governed predominantly by diffusion-controlled processes rather than by immobilization-limited electron transfer [29].

**Figure 4:**
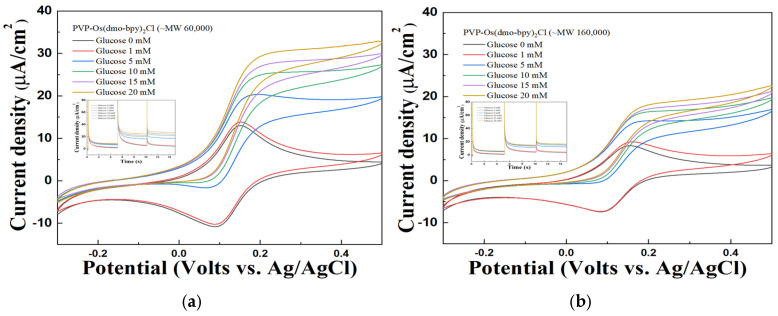
Cyclic voltammogram of different glucose concentrations of PVP-Os(dmo-bpy)_2_Cl (MW 60,000 (**a**)/160,000 (**b**)). (inset) The MPS results of PVP-Os(dmo-bpy)_2_Cl (MW 60,000 (**a**)/160,000 (**b**)) for the detection of various glucose concentrations(0–20 mM) in 1× PBS.

**Figure 5:**
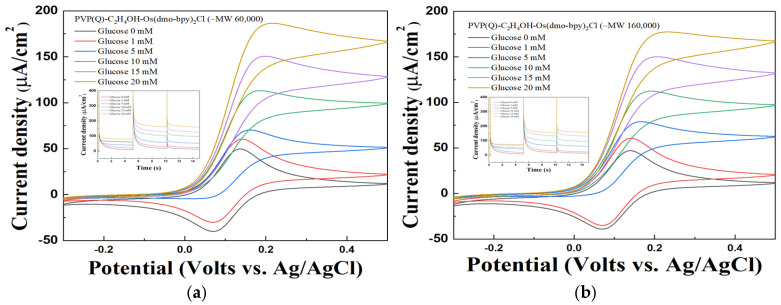
Cyclic voltammogram of different glucose concentrations of PVP(Q)-C_2_H_4_OH-Os(dmo-bpy)_2_Cl (MW 60,000 (**a**)/160,000 (**b**)). Inset: The MPS results of PVP(Q)-C_2_H_4_OH-Os(dmo-bpy)_2_Cl (MW 60,000 (**a**)/160,000 (**b**)) for the detection of various glucose concentrations(0–20 mM) in 1× PBS.

The performance of the synthesized mediators as electron transfer mediators was evaluated using CV and MPS measurements with electrodes modified with PVP(Q)-C_2_H_4_OH-Os(dmo-bpy)_2_Cl/GDH/SPCEs at various glucose concentrations (1, 5, 10, 15, and 20 mM in 1× PBS). The electrodes fabricated with mediators synthesized from non-quaternized PVP exhibited only weak and unstable catalytic current responses toward glucose, as shown in Figure 4. In contrast, the electrodes incorporating quaternized PVP(Q)-C_2_H_4_OH-Os(dmo-bpy)_2_Cl displayed distinct and stable catalytic currents across the same glucose concentrations (Figure 5).

**Figure 6:**
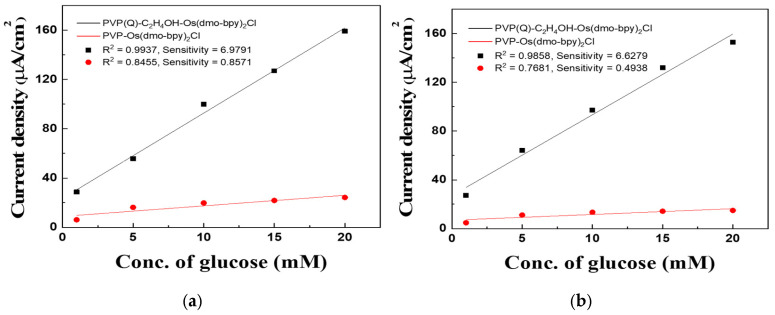
(**a**) Calibration curve between PVP(Q)-C_2_H_4_OH-Os(dmo-bpy)_2_Cl (MW 60,000) (Black line) and PVP-Os(dmo-bpy)_2_Cl (MW 60,000) (Red line) at 0.2 V. (**b**) Calibration curve between PVP(Q)-C_2_H_4_OH-Os(dmo-bpy)_2_Cl (MW 160,000) (Black line) and PVP-Os(dmo-bpy)_2_Cl (MW 160,000) (Red line) at 0.2 V.

The calibration curves derived from MPS measurements at 0.2 V for 5 s (Figure 6a,b) highlight these differences. For electrodes modified with PVP(Q)-C_2_H_4_OH-Os(dmo-bpy)_2_Cl, the sample with a molecular weight of 60,000 exhibited a correlation coefficient (R^2^) of 0.99388 and a sensitivity of 6.9791 μA·mM^−1^·cm^−2^, whereas the sample with a molecular weight of 160,000 showed an R^2^ of 0.98596 and a sensitivity of 6.6279 μA·mM^−1^·cm^−2^. In contrast, electrodes prepared with PVP-Os(dmo-bpy)_2_Cl of 60,000 molecular weight exhibited an R^2^ of 0.88008 and a sensitivity of 0.8571 μA·mM^−1^·cm^−2^, whereas those with 160,000 molecular weight showed an R^2^ of 0.82342 and a sensitivity of 0.4938 μA·mM^−1^·cm^−2^. In addition, at 20 mM glucose, the catalytic current responses of the quaternized electrodes were approximately 6.6-fold (MW 60,000; 159.24 vs. 24.23 μA·cm^−2^) and 10.3-fold (MW 160,000; 153.03 vs. 14.88 μA·cm^−2^) higher than those of the non-quaternized electrodes, confirming the superior electron transfer efficiency of the quaternized polymer complexes.

The interference effects of common electroactive species present in blood were investigated using the fabricated electrodes by MPS measurements. Representative interferents AA, DA, UA, and serotonin were prepared at 0.1 mM in 1× PBS, while glucose solutions were prepared at 0 mM and 0.1 mM. A 40 μL volume of each solution was applied to the electrode surface for testing.

**Figure 7:**
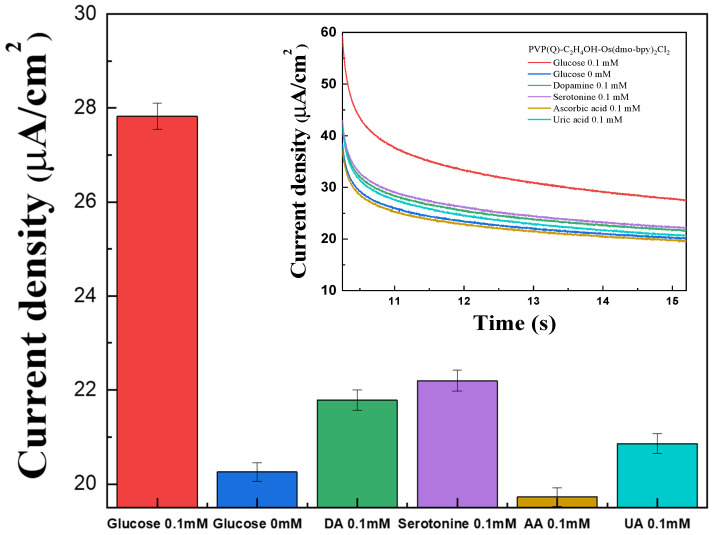
Bar graph for the interference effect of glucose (red), DA (green), serotonin (violet), AA (brown), and UA (cyan) in 1× PBS. (inset) The MPS results for the interference effect of glucose (red), DA (green), serotonin (violet), AA (brown), and UA (cyan) with the PVP(Q)-C_2_H_4_OH-Os(dmo-bpy)_2_Cl (MW 160,000).

The signals generated by AA, DA, UA, and serotonin were higher than those observed for 0 mM glucose, except for AA (Figure 7). On the other hand, all interference responses were significantly lower than the signal obtained for 0.1 mM glucose, which is below typical physiological glucose levels. These results show that the influence of electroactive interferents is negligible across the tested range, confirming the high selectivity of the PVP(Q)-C_2_H_4_OH-Os(dmo-bpy)_2_Cl/GDH/SPCEs toward glucose detection.

### 3.5. Electrochemical Characteristics of PVP(Q)-C_2_H_4_OH-Os(dmo-bpy)_2_Cl/GDH/Silane/Au (MW 160,000)

**Figure 8:**
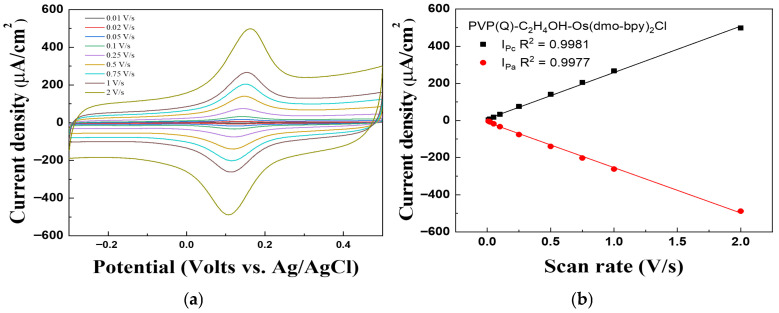
(**a**) Cyclic voltammograms and (**b**) Calibration curve vs. scan rate (0.01–2 Vs^−1^) for the PVP(Q)-C_2_H_4_OH-Os(dmo-bpy)_2_Cl/GDH/Silane/Au electrodes (MW 160,000).

Figure 8 shows the scan rate dependence of the PVP(Q)-C_2_H_4_OH-Os(dmo-bpy)_2_Cl/GDH/Silane/Au electrodes, recorded in the range of 0.01–2 V/s, to evaluate their performance as electron transfer mediators and investigate the diffusion behavior. The cathodic peak current (I_pc_) and anodic peak current (I_pa_) exhibited excellent linearity with the correlation coefficients of R^2^ = 0.9981 and R^2^ = 0.9977, respectively.

The current density increased proportionally with the scan rate, indicating that the electron transfer mediator and enzyme were chemically immobilized on the Au electrode. This behavior confirms that the electrochemical process is governed by immobilization on the electrode surface rather than diffusion in solution, showing the characteristics of a surface-confined electrode [30].

**Figure 9:**
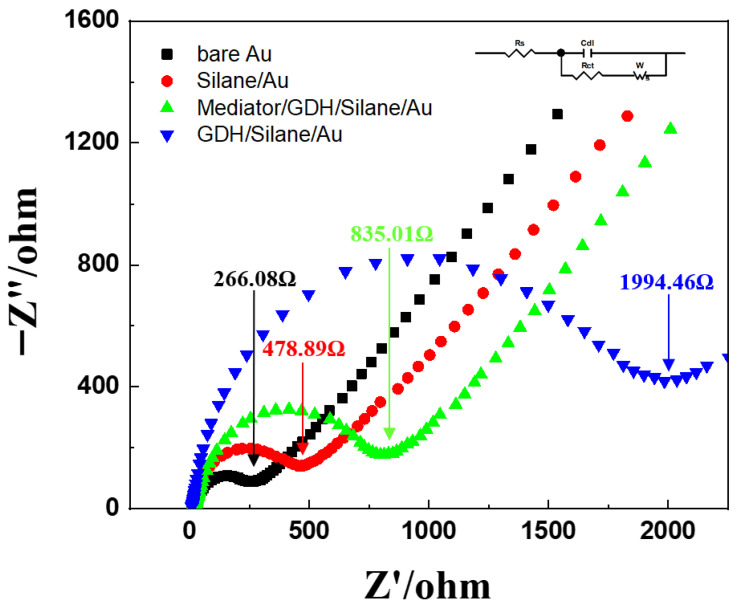
Electrochemical impedance spectroscopy (EIS) of the bare Au electrode (black), silane-treated Au electrode (red), Mediator/GDH/Silane/Au electrode (green), and GDH/Silane/Au electrode (blue).

Figure 9 presents the electrochemical impedance spectroscopy (EIS) results of the electrodes at different immobilization stages. EIS was conducted in an electrolyte solution containing 2.0 mM potassium hexacyanoferrate (II) trihydrate and 2.0 mM potassium hexacyanoferrate (III) in 0.5 M KCl (1:1 ratio).

The bare Au electrode showed the lowest charge transfer resistance (R_ct_) of 266.08 Ω because of the intrinsic conductivity of Au. In contrast, the silanized Au electrode showed an increased R_ct_ of 478.89 Ω, reflecting the additional resistance introduced by the self-assembled monolayer. A further increase was observed for the GDH/Silane/Au electrode, with an R_ct_ of 1994.46 Ω, which can be attributed to the insulating nature and large molecular size of the enzyme layer that hindered electron transfer. In particular, the Mediator/GDH/Silane/Au electrode showed a significantly reduced R_ct_ of 835.01 Ω compared to the GDH/Silane/Au electrode, indicating that the immobilized mediator facilitated electron transfer.

These results show that the mediator was effectively immobilized on the Au electrode surface through chemical bonding, enhancing the stability and efficiency of electron transfer [31].

**Figure 10:**
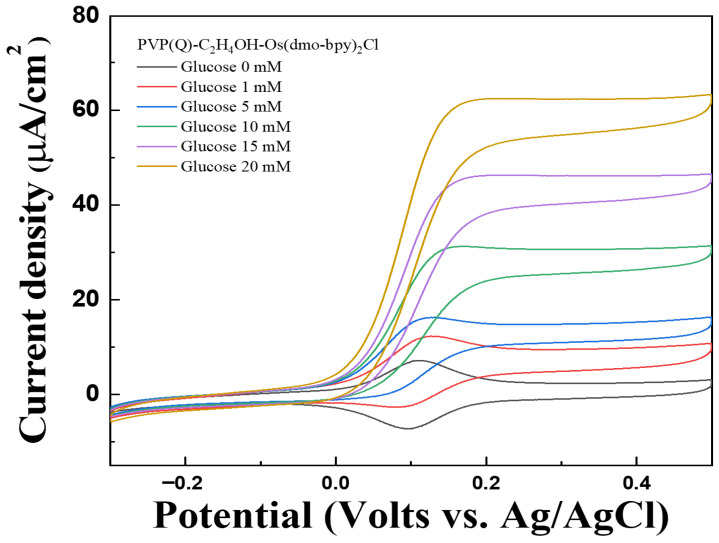
Cyclic voltammograms (vs. Ag/AgCl) of Au electrodes/GDH/Silane/PVP(Q)-C_2_H_4_OH-Os(dmo-bpy)_2_Cl (MW 160,000) for the detection of various glucose concentrations (0–20 mM) in 1× PBS.

**Figure 11:**
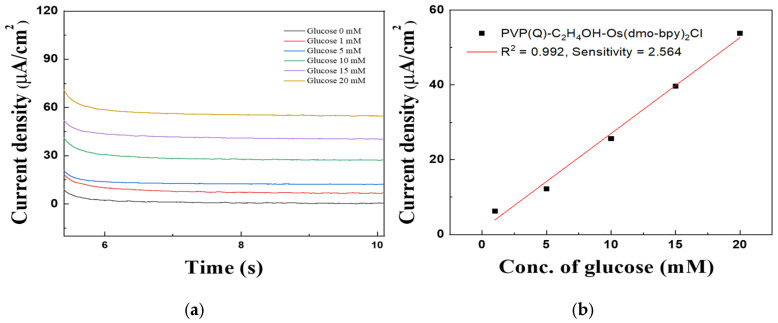
(**a**) MPS results of Au electrodes/GDH/Silane/PVP(Q)-C_2_H_4_OH-Os(dmo-bpy)_2_Cl (MW 160,000) for the detection of various glucose concentrations (0–20 mM). (**b**) Calibration curve of the corresponding relationship between current density and glucose concentration at 0.2 in (**a**).

The performance of PVP(Q)-C_2_H_4_OH-Os(dmo-bpy)_2_Cl as an electron transfer mediator was assessed using CV and MPS measurements at various glucose concentrations (0, 1, 5, 10, 15, and 20 mM in 1× PBS) using PVP(Q)-C_2_H_4_OH-Os(dmo-bpy)_2_Cl/GDH/Silane/Au electrodes (MW 160,000). The electrodes exhibited distinct and stable catalytic current responses across the tested glucose concentrations, as shown in Figure 10 and Figure 11a.

The calibration curve obtained from MPS measurements at 0.2 V for 5 s (Figure 11b) showed excellent linearity in the glucose concentration range of 1–20 mM. The correlation coefficient (R^2^) was 0.992, and the sensitivity was calculated to be 2.564 μA·mM^−1^·cm^−2^, confirming the high electrochemical responsiveness of the mediator-immobilized electrodes.

**Figure 12:**
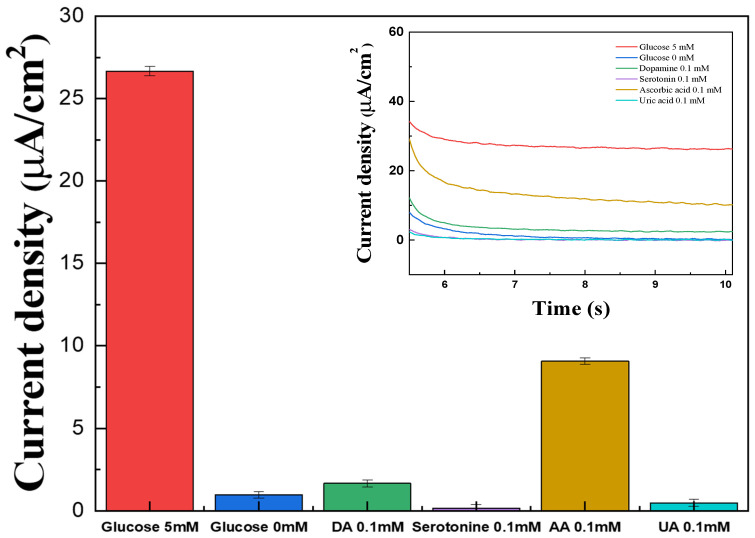
Bar graph showing the interference effect of glucose (red), DA (green), serotonin (violet), AA (brown), and UA (cyan) in 1× PBS. (inset) The MPS results for the interference effect of glucose (red), DA (green), serotonin (violet), AA (brown), and UA (cyan) with the PVP(Q)-C_2_H_4_OH-Os(dmo-bpy)_2_Cl/GDH/Silane/Au electrode (MW 160,000).

Figure 12 presents the results of MPS measurements performed to examine the interference effects of common electroactive species under physiological conditions. Representative interferents (AA, DA, UA, and serotonin) were each prepared at 0.1 mM in 1× PBS, while glucose solutions were prepared at 0 mM and 5 mM. A 15 μL volume of each solution was applied to the electrode surface for analysis.

The current responses generated by DA, UA, and serotonin were comparable to those observed in the absence of glucose and were significantly lower than the signal observed for 5 mM glucose, corresponding to typical blood glucose levels (Figure 12). Although AA showed a slightly stronger response, its contribution was still negligible compared to glucose. These results show that the fabricated electrodes exhibit high selectivity toward glucose, with negligible interference from electroactive species across the tested range.

### 3.6. Long-Term Test and Serum Sample Test

**Figure 13:**
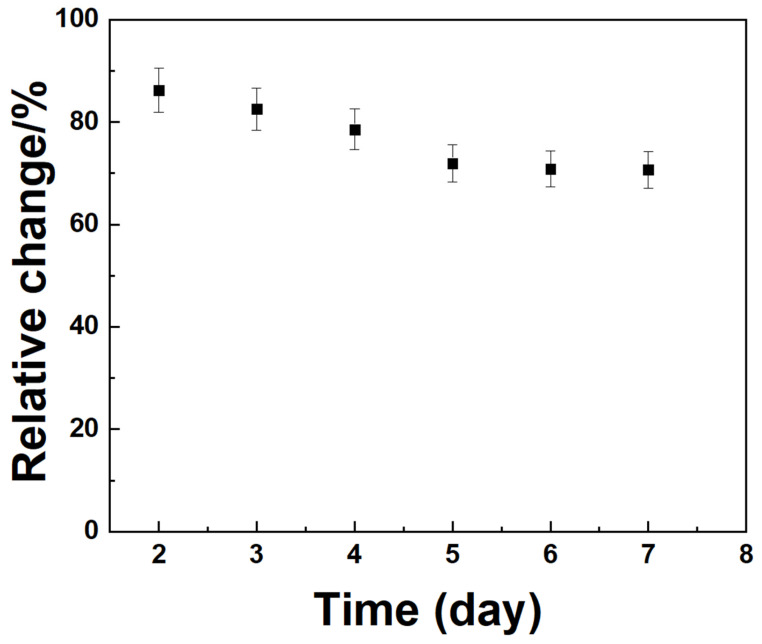
Amperometric I-t curve with I_pa_ for long-term at glucose 5 mM for seven days.

Figure 13 shows the amperometric I–t responses obtained to evaluate the long-term stability of the Mediator/GDH/Silane/Au electrodes for CGM. The measurements were taken in a 5 mM glucose solution over seven days. The results showed that the oxidation current decreased by approximately 18% from the initial enzyme activity to that measured on day 7, suggesting that the electrodes retained a substantial portion of their catalytic activity and showed good operational stability over the testing period.

**Figure 14:**
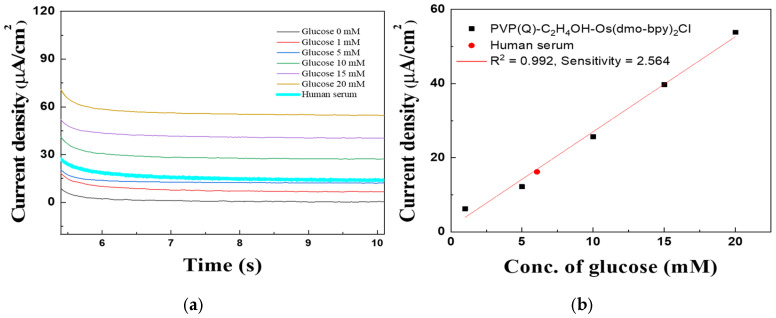
(**a**) MPS results and (**b**) Calibration curve of human plasma to PVP(Q)-C_2_H_4_OH-Os(dmo-bpy)_2_Cl/GDH/Silane/Au electrode (MW 160,000).

Figure 14 shows the MPS results obtained to evaluate the quantitative glucose response of PVP(Q)-C_2_H_4_OH-Os(dmo-bpy)_2_Cl/GDH/Silane/Au electrodes (MW 160,000) in human plasma. A 15 μL aliquot of human plasma was applied to the electrode surface for the measurements. The glucose concentration in the serum was determined using the external standard method. A linear regression equation of *Y* = 2.56*X* + 1.37 was obtained for the PVP(Q)-C_2_H_4_OH-Os(dmo-bpy)_2_Cl-modified electrodes, and the calculated glucose concentration in human plasma was 6.05 mM.

## 4. Discussion and Conclusions

Structural and electrochemical analyses demonstrated that quaternization of PVP effectively enhanced its role as an electron transfer mediator. FT-IR and ^1^H-NMR spectra confirmed the incorporation of 2-bromoethanol, and the actual polymer-to-monomer ratio (~4.1:1) was slightly lower than the feed ratio. Zeta potential values exceeding +88 mV indicated excellent dispersion stability, validating that quaternization improved aqueous solubility. These results confirmed the structural stability and electrochemical suitability of the synthesized PVP(Q)-C_2_H_4_OH-Os(dmo-bpy)_2_Cl complexes.

Electrochemical characterization revealed distinct differences between quaternized and non-quaternized mediators. CV showed a positive redox potential shift in PVP(Q)-C_2_H_4_OH-Os(dmo-bpy)_2_Cl, attributed to the electron-withdrawing pyridine groups. On SPCE electrodes, quaternized mediators generated strong and stable catalytic currents across 1–20 mM glucose, whereas non-quaternized ones showed weak responses. Calibration results demonstrated 6.6–10.3-fold higher sensitivities and excellent linearity (R^2^ > 0.99), confirming enhanced electron transfer efficiency.

Surface analyses confirmed stable mediator and enzyme immobilization. SEM-EDS detected osmium and oxygen signals from the polymer backbone, while FE-SEM revealed clear surface transitions from bare to silanized Au and enzyme/mediator-modified layers.

Electrochemical studies on Au-based electrodes further supported these results. Unlike SPCEs with diffusion-controlled behavior, Au electrodes exhibited surface-confined electron transfer. EIS revealed a substantial reduction in charge transfer resistance (R_ct_ = 835 Ω) compared to GDH-only electrodes (R_ct_ = 1994 Ω), indicating facilitated electron transfer despite the insulating enzyme layer. The mediator showed high glucose sensitivity (2.564 μA·mM^−1^·cm^−2^) and excellent linearity (R^2^ = 0.992) across physiological concentrations.

In the equivalent circuit model used to interpret the EIS spectra, R_s_ represents the solution resistance, which corresponds to ionic conduction through the electrolyte. The charge-transfer resistance (R_ct_) reflects the interfacial electron-transfer kinetics between the enzyme–mediator layer and the electrode surface, where lower R_ct_ indicates more efficient electron tunneling through the polymeric mediator. The constant phase element (CPE) accounts for the non-ideal double-layer capacitance arising from heterogeneous surface roughness and polymer film morphology, while the Warburg impedance (Z_w_) represents the diffusion of electroactive species through the porous enzyme–mediator layer [32]. The significantly reduced R_ct_ and the nearly unchanged R_s_ suggest that PVP(Q)–C_2_H_4_OH–Os(dmo-bpy)_2_Cl facilitates faster interfacial charge transfer without altering bulk electrolyte properties, confirming the role of quaternization in enhancing electron mobility within the mediator matrix.

In comparison with widely used commercial polymers such as Nafion, poly(vinyl alcohol) (PVA), and chitosan, the quaternized PVP(Q)–C_2_H_4_OH matrix demonstrates clear advantages for enzyme-based electrochemical glucose sensing. Nafion is well-known for its ionic conductivity but can lead to partial enzyme inactivation because of its strong acidity and hydrophobic microdomains [11]. PVA has been studied as a matrix for glucose oxidase immobilization and exhibits good biocompatibility but relatively low intrinsic conductivity and restricted mediator mobility, which limit rapid electron transfer [33]. Chitosan and its hydrogel derivatives provide excellent biocompatibility and film-forming ability; however, they often require additional conductive fillers to overcome poor charge transport [34]. In contrast, the cationic PVP(Q) framework ensures superior aqueous solubility, high zeta potential (>+88 mV), and strong electrostatic attraction with the negatively charged GDH enzyme, thereby maintaining homogeneous mediator dispersion and efficient redox communication. These synergistic properties result in markedly enhanced sensitivity (up to ~10×) and operational stability compared with conventional polymer matrices.

Although theoretical calculations were not conducted in this study, the improvement in electron-transfer behavior by quaternization can be explained based on previously reported experimental and mechanistic insights. Aoki et al. demonstrated that partial quaternization of poly(4-vinylpyridine) in osmium-based redox hydrogels significantly increased the apparent electron diffusion coefficient and eliminated its pH dependence, confirming that positively charged pyridinium sites promote faster charge transport through enhanced ionic mobility and electrostatic coupling with redox centers [35]. Similarly, Mavronasou et al. showed that quaternized pyridine frameworks exhibit higher dielectric constants and charge delocalization due to strong solvation of the cationic groups, which facilitates electron tunneling and redox reversibility in conductive polymers [22]. These findings support our electrochemical results, indicating that PVP(Q)–C_2_H_4_OH–Os(dmo-bpy)_2_Cl achieves lower R_ct_ and improved catalytic currents through strengthened polymer–metal electronic interactions.

Selectivity and stability tests verified reliable operation in biological conditions. Negligible interference from AA, DA, UA, and serotonin ensured specificity, and the electrodes retained ~82% of initial current after seven days. Human plasma testing yielded 6.05 mM glucose, consistent with physiological levels, confirming clinical feasibility.

Although all electrochemical measurements were performed under static conditions in phosphate-buffered saline, the observed reproducibility and stability suggest that the quaternized polymeric mediator maintains its electrochemical activity even in complex biological environments. In practical CGM applications, dynamic flow and continuous glucose variation could influence mass transport and enzyme–mediator coupling. Moreover, while the seven-day operational stability demonstrated promising robustness, longer-term evaluation is essential for continuous monitoring applications that typically require multi-week use. Future work will therefore involve extended amperometric tests under physiological conditions, accompanied by post-measurement spectroscopic verification of mediator integrity and enzyme activity retention. These studies will clarify the long-term durability and reliability of the PVP(Q)–C_2_H_4_OH–Os(dmo-bpy)_2_Cl system for real-time CGM operation. Future studies will therefore focus on evaluating the in situ performance of the PVP(Q)–C_2_H_4_OH–Os(dmo-bpy)_2_Cl mediator under simulated physiological flow systems and microfluidic platforms to verify its long-term operational reliability.

In conclusion, PVP quaternization markedly improved solubility and electron transfer of osmium-based mediators. The resulting PVP(Q)-C_2_H_4_OH-Os(dmo-bpy)_2_Cl complexes exhibited superior electrochemical performance, selectivity, and durability on Au electrodes. Accurate glucose detection in human plasma demonstrates their strong potential for next-generation CGM applications, effectively linking polymer design with real-world biosensor performance.

## Data Availability

The original contributions presented in this study are included in the article and Supplementary Materials. Further inquiries can be directed to the corresponding authors.

## Supplementary Materials

The following supporting information can be downloaded at: https://www.mdpi.com/article/10.3390/polym17212874/s1, Figure S1. FT-IR spectrum PVP(Q)-C_2_H_4_OH (~MW 60,000), PVP(Q)-C_2_H_4_OH (~MW 160,000), 2-Bromoethanol, PVP (~MW 60,000), and PVP (~MW 160,000); Figure S2. ^1^H-NMR spectrum of PVP(Q)-C_2_H_4_OH-Os(dmo-bpy)_2_Cl (MW 60,000: (a); 160,000: (b)); Figure S3. Zeta-potential of PVP(Q)-C_2_H_4_OH-Os(dmo-bpy)_2_Cl (MW 60,000 Black line), PVP(Q)-C_2_H_4_OH-Os(dmo-bpy)_2_Cl (MW 160,000 Red line); Figure S4. Cyclic voltammogram (vs. Ag/AgCl) of Os(dmo-bpy)_2_Cl_2_ (red line), PVP(Q)-C_2_H_4_OH-Os(dmo-bpy)_2_Cl (blue line); Figure S5. SEM(a) and EDS(b) of Mediators@GDH@X-linker/SPCEs.

## Abbreviations

The following abbreviations are used in this manuscript:

CGM	Continuous glucose monitoring
GDH	Glucose dehydrogenase
GOx	Glucose oxidase
BGM	Blood glucose monitoring
PEGDGE	Poly(ethylene glycol) diglycidyl ether
AA	Ascorbic acid
DA	Dopamine hydrochloride
UA	Uric acid
3-GOPTS	(3-glycidyloxypropyl)trimethoxysilane
FAD	Flavin adenine dinucleotide
SPCEs	Screen-printed carbon electrodes
OHP	Overhead project
CV	Cyclic voltammetry
MPS	Multi-potential step
PVP	Poly(4-Vinylpyridine)
PVP(Q)	Quaternized Poly(4-Vinylpyridine)
