# Supplementary material for: Enhanced Solubility and Electron Transfer of Osmium-Based Mediators via Quaternized Poly(4-Vinylpyridine) for Electrochemical Glucose Detection
Source: Polymers (Basel). 2025 Oct 28;17(21):2874. doi: 10.3390/polym17212874 (PMC12610834; doi:10.3390/polym17212874)
Supplement: Supplementary file 1 [file polymers-17-02874-s001.zip › polymers-3925013-supplementary.pdf]

polymers

MDPI

Type of the Paper (Article)

Supplementary Information

Enhanced Solubility and Electron Transfer of Osmium-Based Mediators via Quaternized Poly(4-vinylpyridine) for Electrochemical Glucose detection

Yun Yeong Cho [1†], Tae-Won Seo [1†], Young-Bong Choi [1,2*] and Won-Yong Jeon [3,4,5*]

1 Department of Chemistry, College of Science & Technology, Dankook University, 119 Dandae-ro, Dongnam-gu, Cheonan-si 31116, Chungnam, Republic of Korea
2 Department of Cosmedical Materials, College of Bio-Convergence, Dankook University, 119 Dandae-ro, Dongnam-gu, Cheonan-si 31116, Chungnam, Republic of Korea
3 Graduate School of Management of Technology, Hoseo University, Asan 31499, Chungnam, Republic of Korea
4 Fine Dust & Net Zero Research Institute, Hoseo University, Asan 31499, Chungnam, Republic of Korea
5 Eco Upcycling R&D Center, Hoseo University, Asan 31499, Chungnam, Republic of Korea

* Authors to whom correspondence should be addressed.: chem0404@dankook.ac.kr and powerwy@hoseo.edu
† These authors contributed equally to this work.

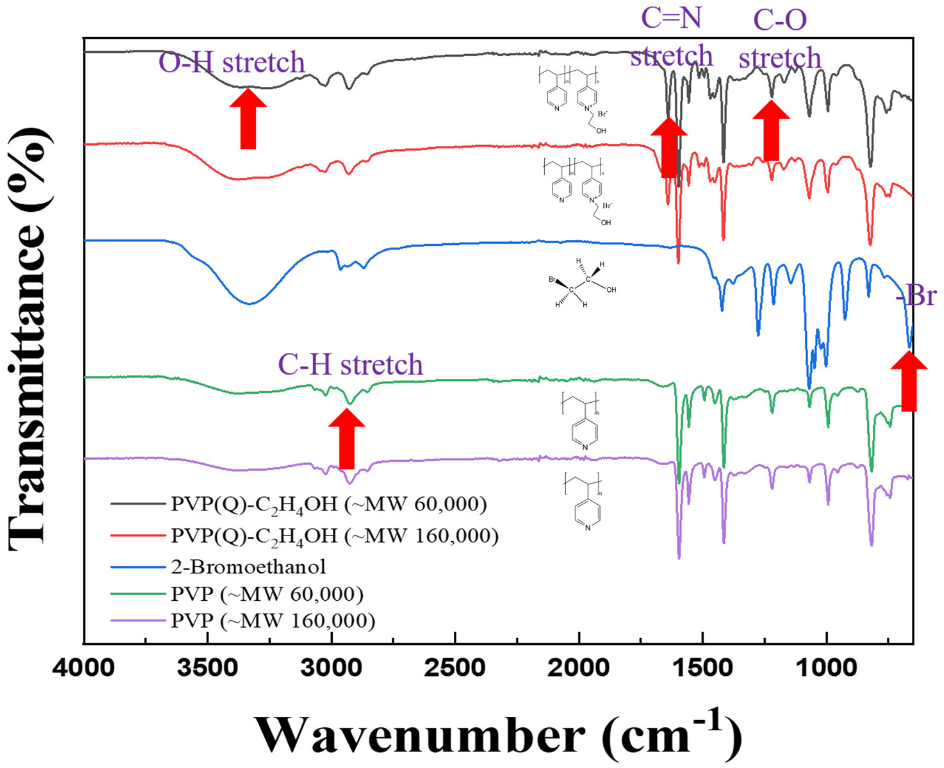

Figure S1. FT-IR spectrum PVP(Q)-C_2H_4OH (~MW 60,000), PVP(Q)-C_2H_4OH (~MW 160,000), 2-Bromoethanol, PVP (~MW 60,000), and PVP (~MW 160,000).

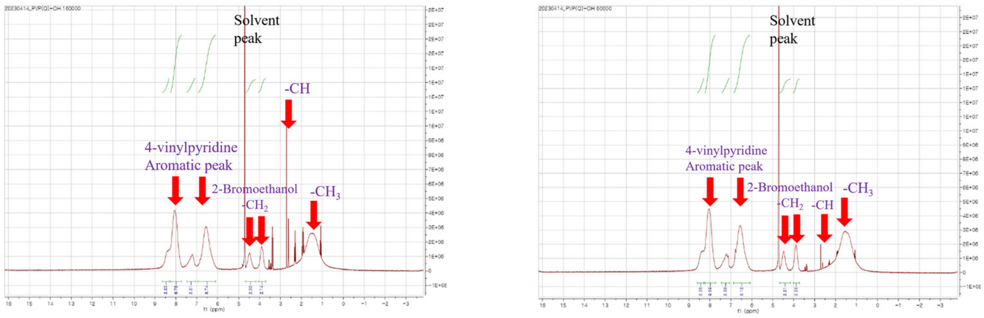

(**a**) (**b**)

Figure S2. ^{1}H-NMR spectrum of PVP(Q)-C_2H_4OH-Os(dmo-bpy)$_2$Cl (MW 60,000: (a); 160,000: (b)).

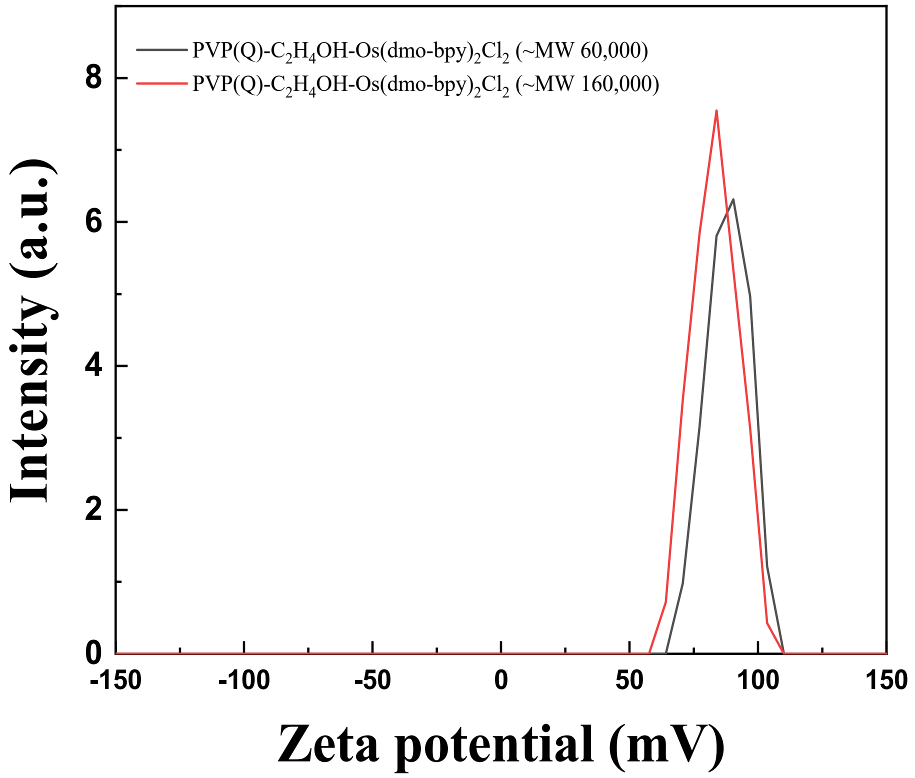

Figure S3. Zeta-potential of PVP(Q)-C_2H_4OH-Os(dmo-bpy)$_2$Cl (MW 60,000 Black line), PVP(Q)-C_2H_4OH-Os(dmo-bpy)$_2$Cl (MW 160,000 Red line).

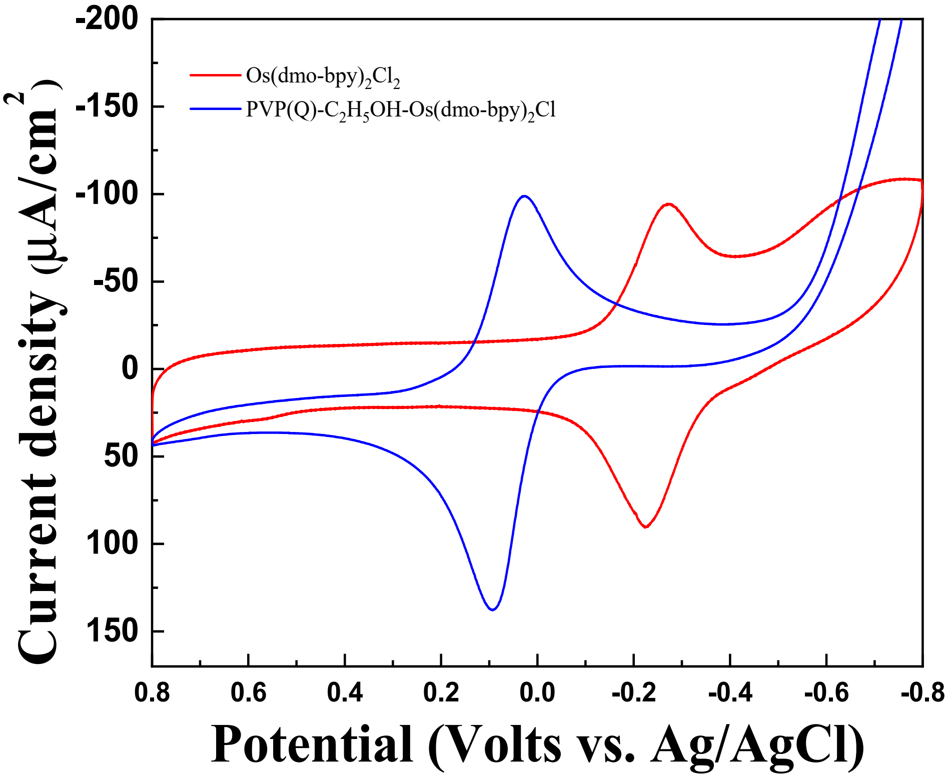

Figure S4. Cyclic voltammogram (vs. Ag/AgCl) of Os(dmo-bpy)$_2$Cl$_2$ (red line), PVP(Q)-C$_2$H$_4$OH-Os(dmo-bpy)$_2$Cl (blue line).

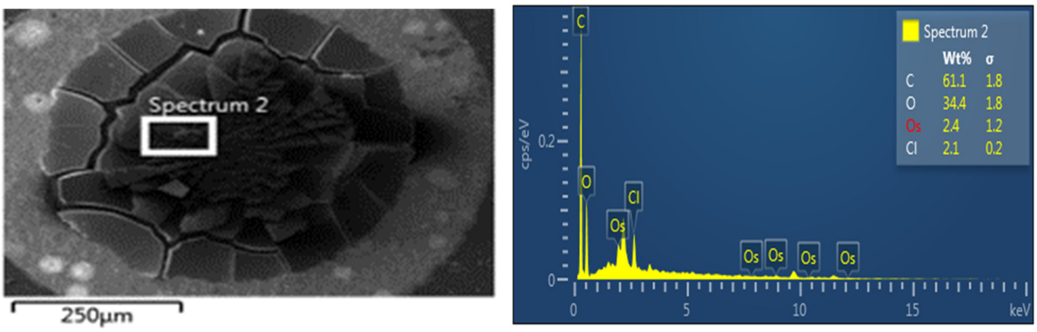

(a) (b)

Figure S5. SEM(a) and EDS(b) of Mediators@GDH@X-linker/SPCEs
